# Changes in the Epidemiology of Zoonotic Infections in Children: A Nationwide Register Study in Finland

**DOI:** 10.1097/INF.0000000000003440

**Published:** 2021-12-28

**Authors:** Ilari Kuitunen, Marjo Renko

**Affiliations:** From the * Institute of Clinical Medicine, Department of Pediatrics, University of Eastern Finland, Kuopio; † Department of Pediatrics, Mikkeli Central Hospital, Mikkeli; ‡ Department of Pediatrics, Kuopio University Hospital, Kuopio, Finland.

**Keywords:** zoonosis, *Borrelia burgdorferi*, Puumala virus, *Francisella tularensis*, tick-borne encephalitis virus

## Abstract

Zoonotic infections are difficult to recognize in children. The age distributions and seasonal occurrences of these infections vary substantially, even among those transmitted by the same vectors, and their epidemiology may change over time. The aim was to report the incidences and trends of *Borrelia burgdorferi*, Puumala virus, *Francisella tularensis* and tick-borne encephalitis (TBE) virus infections in the pediatric population (age 0–19) of Finland.

**Methods:**

A nationwide survey based on the National Infectious Disease Register was conducted from 1996 to 2019 and all laboratory-confirmed cases were included. Age-stratified incidences per 100,000 person-years were calculated.

**Results:**

Cumulative incidences were *B. burgdorferi* 11.2, TBE 0.4, Puumala virus 6.4 and *F. tularensis* 2.5 per 100,000 person-years. An increasing trend in the incidences of *B. burgdorferi* and TBE was observed. Borrelia expanded geographically northward and inland. Tularemia follows a 2–4-year epidemic cycle and rates are similar across age groups. Puumala incidences are highest in the older children.

**Discussion:**

Borrelia infections increased most rapidly in children 5–9 years of age and overall expanded geographically in Finland. Tularemia epidemic cycles were shorter than those previously reported. These results will help clinicians to identify these infections in different geographic areas and age groups in Finland.

## Abstract 2

Supplemental Digital Content is available in the text.

Monitoring zoonotic infections is a crucial part of monitoring public health, and awareness of the epidemiology of these infections can help to identify potential hazards.^1^ Clinicians benefit from the knowledge of the current epidemiological situation, as it guides the prior probability and the predictive values of diagnostic tests.^2,3^ Globally, 70% of currently emerging infectious diseases are zoonotic.^4,5^ Historical studies have shown that climate change has changed global pathogen distributions. According to predictions based on modeling, the rates of zoonotic infections (including vector-borne diseases) are expected to increase with climate change.^6–8^ The overall rate of zoonotic infections in Finland is low, and the most common zoonotic infections in Finland are Puumala virus, *Borrelia burgdorferi* and *Francisella tularensis* infections.^9,10^

Puumala virus is a European hantavirus that causes regional outbreaks of hemorrhagic fever with renal syndrome (nephropathia epidemica) in humans. The reservoir of the virus is bank vole and humans get infected by inhaling secretions of the rodent.^11^ Typical symptoms of nephropathia epidemica include fever, nausea, abdominal pain and headache, and the severity of these symptoms increases with age. Severe forms of renal syndromes have only been detected in adults, and the disease burden is higher in adults.^12,13^

*B. burgdorferi* infections (Lyme disease) are the most common tick-borne infections in Scandinavia.^14^ The natural hosts of *B. burgdorferi* are small mammals, and ticks of the genus *Ixodes* carry this spirochetal bacterium from small mammals to humans. Early infection with this pathogen may present as localized skin rash (erythema migrans), then proceed to early disseminated infection and finally to late infection. Erythema migrans is diagnosed clinically, and the other stages of *B. burgdorferi* infection are diagnosed by serology.^15^ The incidence of laboratory-confirmed *B. burgdorferi* infection has increased in Finland. *B. burgdorferi* infections are transmitted by ticks, and their geographic distribution in Finland has expanded over time.^16^

The tick-borne encephalitis (TBE) virus belongs to the flaviviruses and is endemic to Finland.^17^ In 2019, the incidence of TBE in Finland was 1.7 per 100,000 persons.^10^ The incidence of TBE in Finland has increased and is expected to continue to increase in the near future,^18,19^ as its geographic distribution is expanding northward due to climate warming.^19^ The TBE virus is transmitted from its natural hosts (eg, birds and large mammals, such as deer, hares, foxes and even domestic livestock) to humans by *Ixodes ricinus* ticks. Accordingly, the incidence of TBE has been associated with the density of white deer in Finland.^18^

*F. tularensis* is a Gram-negative intracellular bacterium that most frequently occurs as an ulceroglandular infection in humans.^20^ *F. tularensis holarctica* (type B) is endemic to Finland,^31^ and its most common natural hosts in Finland are small rodents and hares. *F. tularensis* is typically transmitted by mosquitos in Scandinavia, whereas ticks are its main vectors in the United States.^21^ The annual incidences of *F. tularensis* infection have exhibited high variation, as *the* epidemics have been observed to peak every 7–9 years.^22^ In children, the most common symptoms of tularemia are primary skin ulcers and swollen lymph nodes often accompanied with fever.

The aim of the present study was to report the changes in the epidemiology of *B. burgdorferi*, TBE virus, *F. tularensis* and Puumala virus infections during the last 24 years in the pediatric population of Finland.

## MATERIALS AND METHODS

Data for this retrospective nationwide register-based study were mainly gathered from the National Infectious Disease Register, which is maintained by the Finnish Institute of Health and Welfare. The National Infectious Disease Register was established in 1994 and contains information concerning laboratory-confirmed pathogen findings. The entire list of included pathogens reported to laboratories is presented in the register description.^10^ Laboratories in Finland are mandated by Finnish law to report any positive findings to the register. The typical reporting delay is less than one month. We gathered the information on clinically diagnosed erythema migrans cases of *B. burgdorferi* from the open-access report of the Care Register for Primary Health Care, which is a nationwide primary care outpatient register. It holds information on the visit rates and given diagnoses. The Care Register has followed clinically diagnosed *B. burgdorferi* cases since 2011.

For the present study, we collected the monthly numbers of laboratory-confirmed zoonotic infections in humans reported to the infectious disease register from January 1996 to December 2019. The pathogens included in this study were *B. burgdorferi*, TBE virus, *F. tularensis* and Puumala virus. The diagnoses included were all based on serological findings and the inclusion criteria have generally been a 4-fold increase in antibody levels or a prespecified threshold limit. Unfortunately, the data does not include specific criteria for each year and therefore these are not presented alongside the results. We collected the yearly number of clinically diagnosed *B. burgdorferi* cases (erythema migrans) from the Care Register from 2011 to 2019. Additionally, open-access climate data from the Finnish Meteorological Institute was gathered for the study period to be presented as the figure for discussion.^23^

### Data Availability Statement

All data reported in this study are available upon request from the corresponding author.

### Statistics

Yearly incidences of laboratory-confirmed infections and clinically diagnosed *B. burgdorferi* cases were calculated per 100,000 children and are reported per 100,000 person-years per age group. In the incidence calculations, the yearly number of diagnoses in a certain age group was divided by the population number at the beginning of the year in this age group and then multiplied by 100,000 to present the incidence per 100,000 person-years. For the geographic analysis of *B. burgdorferi*, the study period was divided into three equally long periods (8 years). The incidences in these 8-year periods were calculated by dividing the cumulative numbers reported during the 8 years in a certain age group by the sum of the corresponding population and then multiplying the result by 100,000 to present the incidence per 100,000 person-years. In the register, the data are available in 5-year age groups (0–4, 5–9, 10–14 and 15–19 years of age). The yearly pediatric population of these age groups was gathered from the official population reports of Statistics Finland.^24^ All statistical analyses were conducted using SPSS version 27.0 (IBM Corp., Armonk, NY).

## RESULTS

### Overall

**FIGURE 1.**
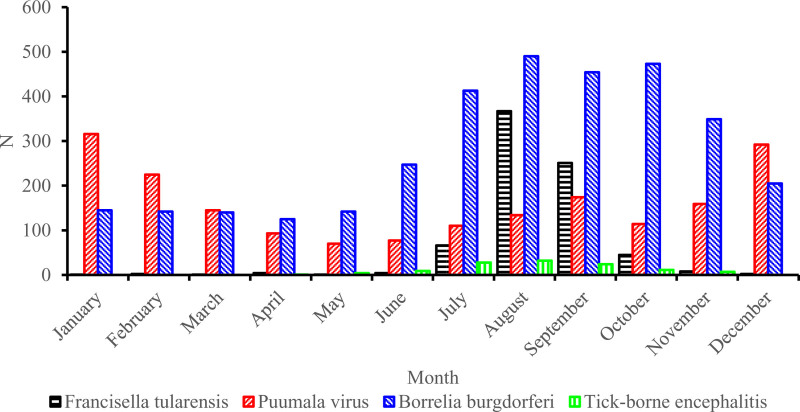
Cumulative numbers and monthly distributions of Puumala virus, *Borrelia burgdorferi* and *Francisella tularensis* infections as well as tick-borne encephalitis.

A total of 6107 laboratory-confirmed zoonotic microbial findings were reported to the register for patients 0–19 years of age during the years 1996–2019. Of these findings, 3325 (54%) were cases of *B. burgdorferi*, 1909 (31%) cases of Puumala virus, 752 (12%) cases of *F. tularensis* and 129 (2%) cases of TBE. *B. burgdorferi* infections were detected throughout the year, TBE was mainly detected from May to November, Puumala virus infections were mainly detected in autumn, and *F. tularensis* infections were mainly detected from July to October (Fig. 1). A total of 2945 clinically diagnosed cases of *B. burgdorferi* were reported to the care register from 2011 to 2019.

### Laboratory-confirmed *Borrelia burgdorferi*

**FIGURE 2.**
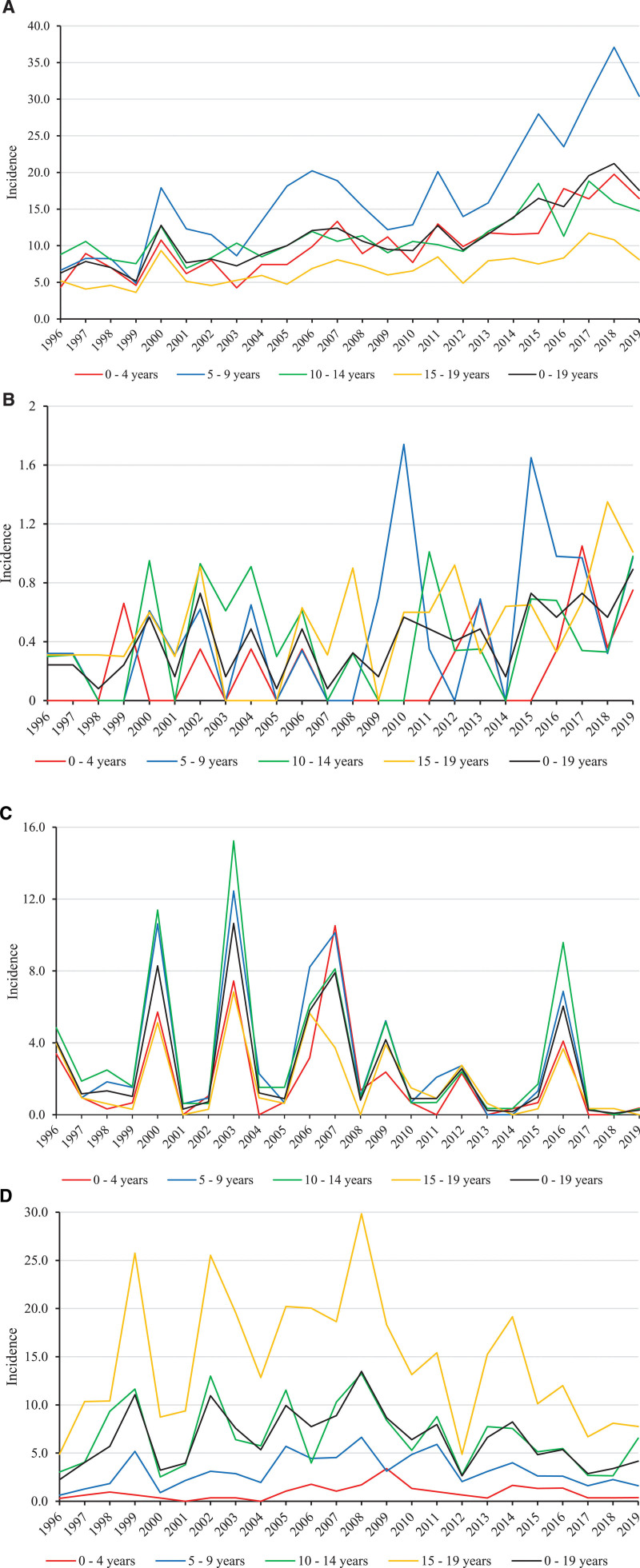
A: Incidences of *Borrelia burgdorferi* infections stratified by age. B: Incidence of tick-borne encephalitis in children stratified by age. C: Incidences of *Francisella tularensis* infections, stratified by age. D: Incidences of Puumala virus infections, stratified by age.

A total of 3325 laboratory-confirmed *B. burgdorferi* cases were diagnosed in patients 0–19 years of age. Of these infections, 725 (22%) were detected in patients 0–4 years, 1248 (37%) in patients 5–9 years, 833 (25%) in patients 10–14 years and 519 (16%) in patients 15–19 years of age. The 24-year cumulative incidence of *B. burgdorferi* infection was 11.2 per 100,000 person-years. The highest recorded incidence of this infection (37.1 per 100,000 person-years) occurred in 2018 among 5–9-year-old patients. The incidence of this infection has exhibited an increasing trend, especially among children 5–9 years of age (Fig. 2A). *B. burgdorferi* infections were mainly found in Southwestern and Southern Finland, and the highest cumulative incidence (51.2 per 100,000 person-years) was reported in the region of Finland Proper. The Åland Islands are part of Finland Proper in Figures, but the cumulative incidence of *B. burgdorferi* infection in the Åland Islands was 525 per 100,000 person-years (Figure, Supplemental Digital Content 1, http://links.lww.com/INF/E612). The geographic distribution of *B. burgdorferi* has expanded inland and toward the northern parts of Finland (Figure, Supplemental Digital Content 2, http://links.lww.com/INF/E613).

### Clinically Diagnosed *Borrelia burgdorferi*

**FIGURE 3.**
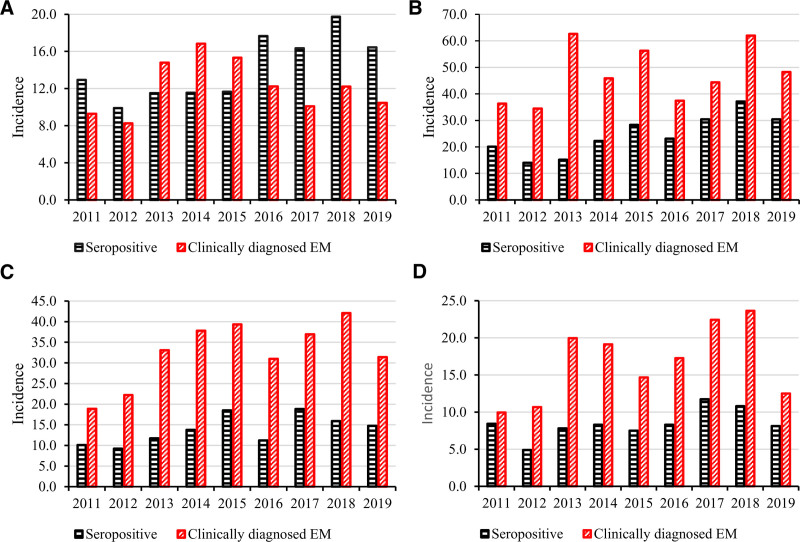
Incidences of seropositive *Borrelia burgdorferi* incidence and clinically diagnosed erythema migrans cases stratified by age (A = 0–4 years, B = 5–9 years, C = 10–14 years, and D = 15–19 years) from 2011 to 2019.

A total of 2945 clinically diagnosed cases (erythema migrans) were reported in patients 0–19 years of age from 2011 to 2019. Of these cases, 322 (11%) were detected in patients 0–4 years, 1294 (44%) in patients 5–9 years, 866 (29%) in patients 10–14 years and 463 (16%) in patients 15–19 years of age. The incidence of clinically diagnosed cases was lower only among patients 0–4 years of age and notably higher in all other age groups (Fig. 3A–D).

### Tick-borne Encephalitis

A total of 129 laboratory-confirmed TBE cases were reported in patients 0–19 years old. Of these cases, 15 (12%) were detected in patients 0–4 years, 40 (31%) in patients 5–9 years, 32 (25%) in patients 10–14 years and 42 (33%) in patients 15–19 years of age. The cumulative incidence of TBE was found to be 0.4 per 100,000 person-years. The highest recorded yearly incidence of TBE (0.9 per 100,000 person-years) occurred in 2019, and the incidence of TBE appeared to exhibit an increasing trend (Fig. 2B). Cases of TBE were mainly found in Southwestern Finland, and the highest regional cumulative incidence (22 per 100,000 person-years) was reported in the Åland Islands, which are part of Finland Proper (Figure, Supplemental Digital Content 1, http://links.lww.com/INF/E612).

### Francisella tularensis

A total of 752 laboratory-confirmed tularemia cases were registered for patients 0–19 years old. Of these cases, 135 (18%) were detected in patients 0–4 years, 229 (30%) in patients 5–9 years, 248 (33%) in patients 10–14 years and 140 (19%) in patients 15–19 years of age. The cumulative incidence of *F. tularensis* infection was found to be 2.5 per 100,000 person-years. The highest recorded incidence of this infection (15.2 per 100,000 person-years) occurred in 2009 among 10–14-year-old patients. The occurrence of tularemia appeared to follow a cycle, as epidemic peaks occurred in the same years across all age groups. These epidemic peaks occurred every 2–4 years. Between high-incidence years, the incidence of *F. tularensis* infection was near zero (Fig. 2C). Cases of tularemia were mainly found in Western and Northwestern Finland, and the highest regional cumulative incidence (11.5 per 100,000 person-years) was reported in the region of Northern Ostrobothnia (Figure, Supplemental Digital Content 1, http://links.lww.com/INF/E612).

### Puumala Virus

A total of 1909 laboratory-confirmed Puumala virus cases were registered for patients 0–19 years of age. Of these cases, 63 (3%) were detected in patients 0–4 years, 226 (12%) were detected in patients 5–9 years, 502 (26%) were detected in patients 10–14 years, and 1118 (59%) were detected in patients 15–19 years old. The cumulative incidence of Puumala virus infection was found to be 6.4 per 100,000 person-years. The highest recorded incidence of this infection (29.8 per 100,000 person-years) occurred in 2008 among 15–19-year-old patients. The yearly incidences of this infection exhibited high variation among older children, whereas the yearly incidences of this infection among 0–4-year-old and 5–9-year-old children remained low and comparatively stable (Fig. 2D). Puumala virus infections were mainly found in Eastern and Northern Finland, and the highest regional cumulative incidence (36.7 per 100,000 person-years) was reported in the region of Southern Savonia (Figure, Supplemental Digital Content 1, http://links.lww.com/INF/E612).

### Yearly Mean Temperatures

The yearly mean temperature deviation calculations showed an increasing trend. Since 2000, only once the mean temperature has been below the average temperature in 2010 (Figure, Supplemental Digital Content 3, http://links.lww.com/INF/E614). The highest recorded deviation from the average temperature was reported in 2015 when in all parts of Finland the temperature was 2 °C higher than average. This increasing trend is seen in all parts of Finland.

## DISCUSSION

According to the present results, the incidences of *B. burgdorferi* infection and TBE have increased over the past 24 years. *F. tularensis* infections exhibited cyclic changes, reaching epidemic peaks every 2–4 years in the pediatric population. Despite the growing incidence of Puumala virus infections in adults, the incidence of Puumala virus cases in the pediatric population has remained relatively stable, although with high variation in the yearly incidences.

The incidences of the various forms of Lyme disease are followed by outpatient diagnostic statistics and seropositive laboratory findings in Finland.^25^ Only seropositive findings were included in the present study before 2011 because clinically diagnosed cases of Lyme disease were not recorded in the register before 2011. The incidence of seropositive *B. burgdorferi* infection has previously been shown to increase, reaching 40 per 100,000 persons in 2019.^10^ In the presently examined pediatric population, the incidence of this infection was highest in 5–9-year-old children and near the incidence in the general population. The reason for this difference between age groups remains unclear. Similar age distribution has been found also in other countries in Europe and in the United States.^26–28^ Based on our register data, the reasons for age distribution can only be speculated. Playing in the grass and woods and the difficulty to notice tick bites might be suggested as the reason for high incidence at the age of 5–9 years. In all other age groups, the incidences of this infection were much lower.

The estimated seroprevalence of *B. burgdorferi* infection in Finland is 4%,^29^ and its geographic distribution in Finland has expanded over time.^16^ This was corroborated by the present longitudinal analysis, as the cumulative regional incidences of this infection increased in northern regions and appeared to move inland. The regional northward expansion of *B. burgdorferi* infection in Finland is likely due to climate warming and the increasing reservoir of *B. burgdorferi* hosts.^30^ Although Finland is relatively long geographically, temperatures have increased in all regions of the country. Therefore, it is expected that the incidence of vector-borne zoonoses will increase in the future as we have already observed in *B. burgdorferi* infections. It must be noted that in our results, the incidence of *B. burgdorferi* in the Åland Islands was approximately a hundred times higher than the cumulative inland incidence. The Åland Islands are located between Finland and Sweden in the Baltic Sea and it is the warmest part of Finland. It also has the highest reported reservoir of tick population in Finland, which most likely explains the high incidence.

The incidence of pediatric TBE has increased in all endemic countries except for Austria, in which TBE vaccination has been introduced successfully.^31^ The present results specifically showed that the incidence of TBE has increased among children in Finland. In Finland, TBE vaccination is offered for citizens older than 3 years in coastal areas with the highest infection risk. Based on our results, the incidence in the coastal areas was notably higher than in inland among pediatric population. The severity of TBE symptoms increases with age; TBE is typically most severe in adults and may be asymptomatic in small children. Therefore, TBE may be underdiagnosed in small children.^32^ According to previous reports, 20% of pediatric TBE patients may have only fever without any neurological symptoms.^33^

The previously reported geographic pattern of Puumala virus infections was also observed among children in the present study as the incidence was highest in Eastern Finland. Puumala virus is transmitted to humans through the aerosolized secretions of bank voles and mice, and the incidence cycles that occur every 3–4 years are dependent on cyclic changes in the bank vole and mouse populations.^34,35^ These epidemic cycles were observed in our report similar to adults. The seroprevalence of Puumala virus IgG antibodies in the Finnish adult population has been estimated at over 10%.^36^ More than half of all Puumala virus patients in the adult population require hospital admission, and the case fatality rate of Puumala virus in Finland has been estimated at 0.08%.^12^ Also, in the Puumala virus, small children have fewer symptoms or may even be asymptomatic. In the present study, the incidence of reported pediatric Puumala virus cases increased with age. The exact reason for the relative mildness of the symptoms in small children is not yet known, but the same phenomenon has been recognized in TBE, hepatitis A and SARS-CoV-2.

The incidence of *F. tularensis* exhibited high yearly variation. The last epidemic peak in Finland occurred in 2016, with an incidence of 13 per 100,000 persons; in 2019, the incidence was 1 per 100,000 persons.^10^ Previously, epidemic peaks have been observed every 7–9 years.^37,38^ In our report, the epidemic peaks occurred every 2–4 years. The reason for the different epidemic cycle in the general population and pediatric population should be further examined. In the epidemic peak of 2016, the incidence of tularemia in children was near that in the general population. Pediatric tularemia cases were mainly found in Northwestern Finland, possibly due to the high rodent population of this region.^39^ The adult hospitalization rate of tularemia has been estimated at 70%, and the case fatality rate at 0.^40^ In children, hospitalization is needed in approximately 40% of the cases, mainly in the youngest age groups.^31^ The majority of *F. tularensis* infections remain asymptomatic, but the seropositivity in the adult population of Finland has been estimated at nearly 2% during high-incidence years.^22^ Tularemia cases are expected to increase in arctic areas as the climate changes.^41^ Whether climate warming modifies the epidemic cycle periods making them more frequent remains unknown.

As the increase in the mean yearly temperatures has been clear and rapid globally, and also in Finland, the possibility of increasing zoonotic infections and novel pathogens must be considered. The host populations of zoonotic pathogens will expand toward north, as already seen in ticks in Finland. Continuous monitoring of host populations and epizootic infections both in animals and humans is needed to manage the evolving situation.^42^ An integrated One Health approach benefits us more than focusing on humans only.^43^

The main strength of the present study was the use of data from the National Infectious Disease Register, which has exhibited excellent coverage of all laboratory-confirmed infections. All laboratories in Finland are mandated by the law to report all findings of about 70 notifiable and other significant pathogens to this register. The register provides up-to-date, regional, age-stratified open-access data, which allowed us to determine and report geographic distributions over time.

The main limitation of the present study was the lack of available patient records. Patient record data would have been beneficial as we would have been able to report the incidences of hospitalizations due to these infections and report possible mortality. Additionally, we were able to include the clinically diagnosed cases of *B. burgdorferi* only from 2011. Furthermore, the incidences reported herein are likely underestimates as the investigated infections are typically only tested for in children with symptoms that are severe enough to warrant physician attendance, whereas the majority of children with these infections may only have minor symptoms or remain symptomless.

## CONCLUSIONS

The incidences of *B. burgdorferi* infections and TBE exhibited increasing trends and northward expansion in 24 years (during 1996–2019). *F. tularensis* infections appeared to follow cyclic changes, with epidemic peaks occurring every 2–4 years in the pediatric population. Puumala virus infections occurred most frequently in older children, likely because most younger children remain asymptomatic; otherwise, the overall incidence of Puumala virus infection has remained stable. These results may help clinicians to recognize zoonotic infections in practice.

## Supplementary Material
