# Supplementary material for: Changes in the Epidemiology of Zoonotic Infections in Children: A Nationwide Register Study in Finland
Source: Pediatr Infect Dis J. 2021 Dec 28;41(4):e113–9. doi: 10.1097/INF.0000000000003440 (PMC8920006; doi:10.1097/INF.0000000000003440)

Supplemental Digital Content 1A. Geographic distribution of seropositive *Borrelia burgdorferi* infections. Presented as the cumulative incidence from 1996 to 2019 per 100,000 person years.


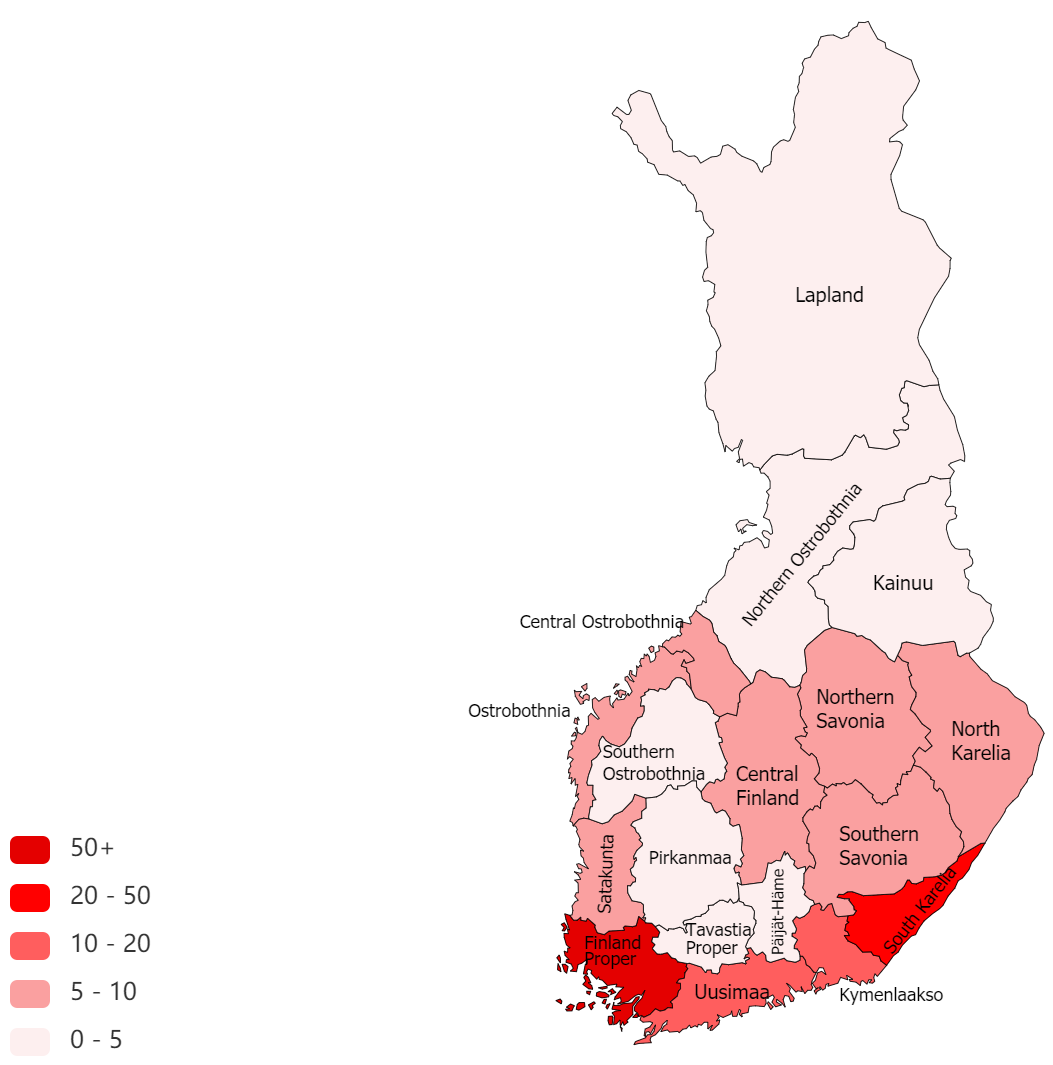


1B. Geographic distribution of tick-borne encephalitis. Presented as the cumulative incidence from 1996 to 2019 per 100,000 person years.


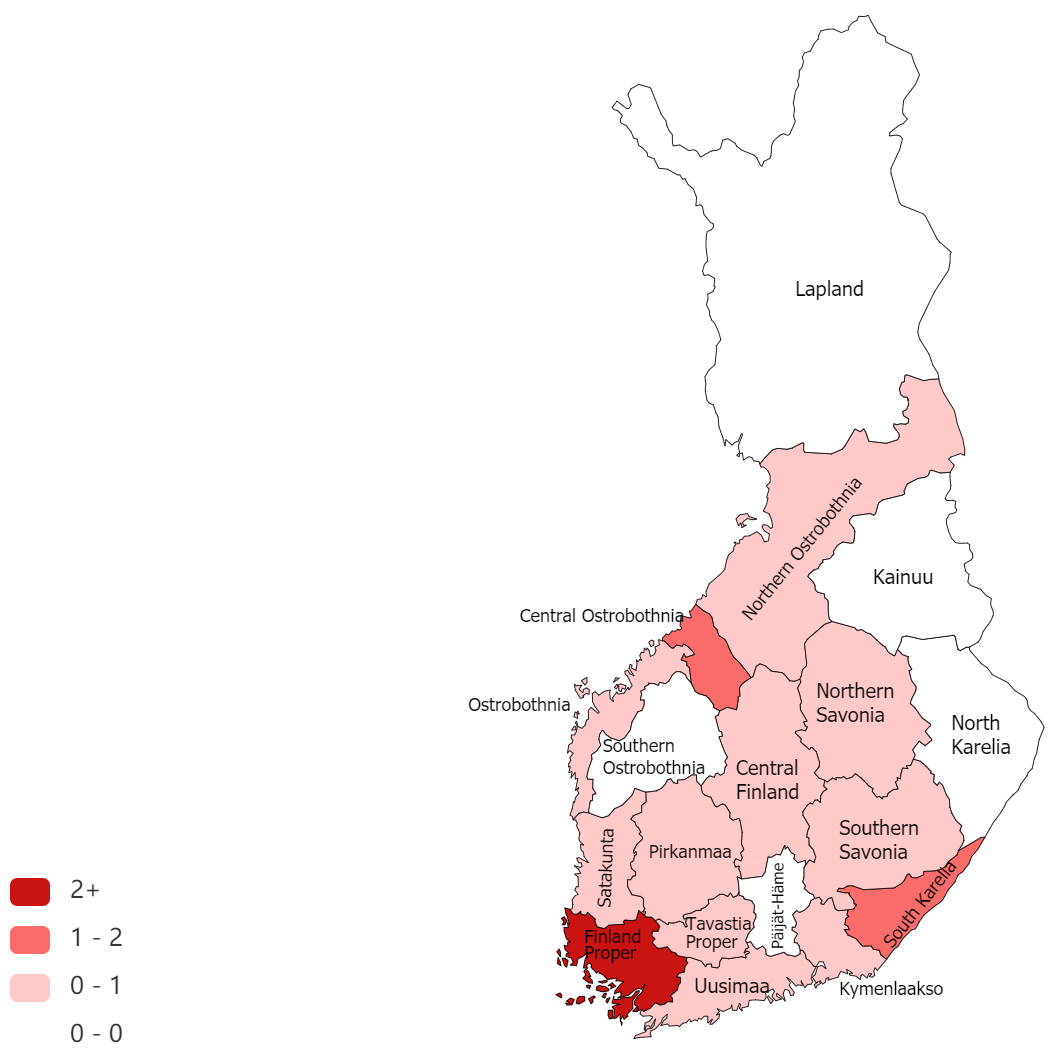


1C. Geographic distribution of *Francisella tularensis* infections in Finland. Presented as the cumulative incidence from 1996 to 2019 per 100,000 person years.


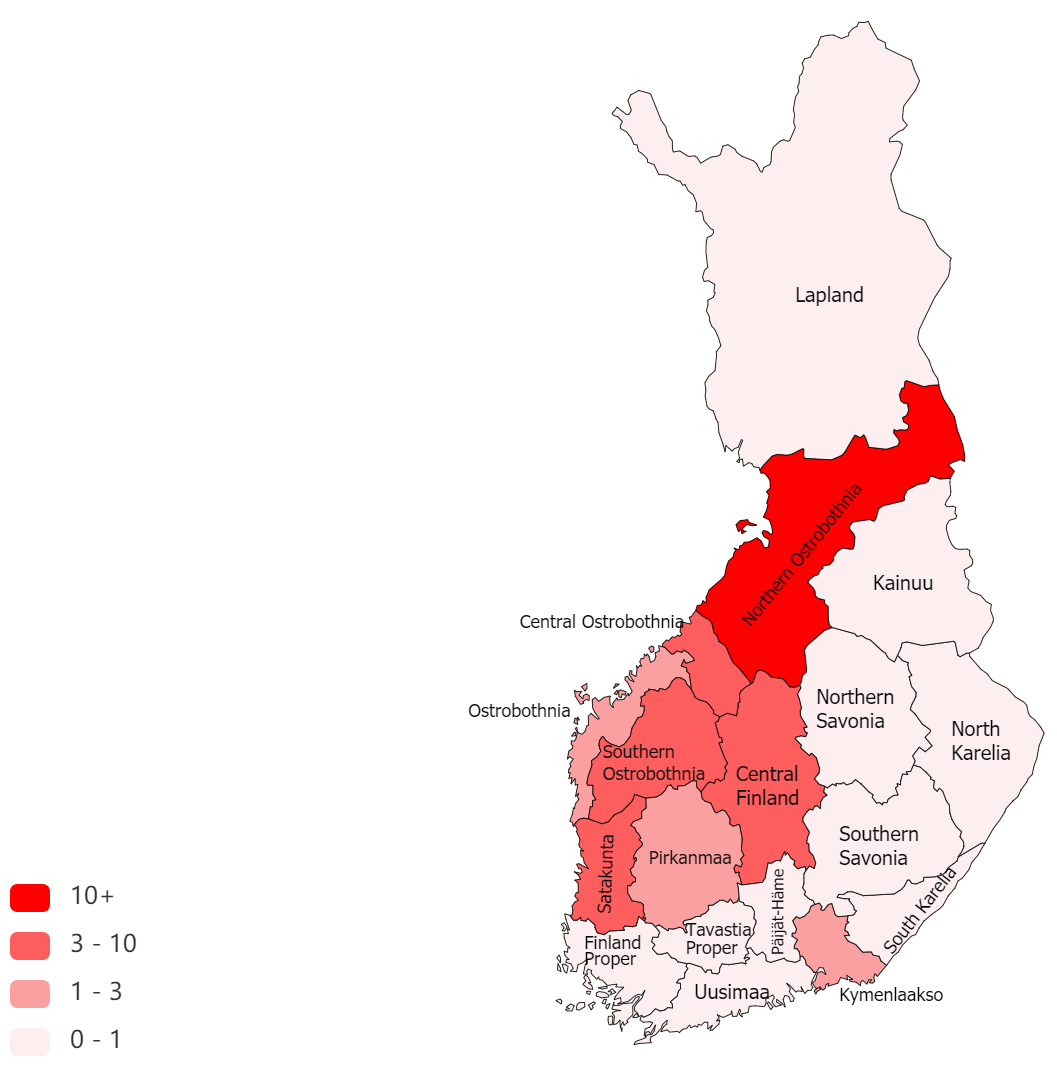


1D. Geographic distribution of Puumala virus infections in Finland. Presented as the cumulative incidence from 1996 to 2019 per 100,000 person years.


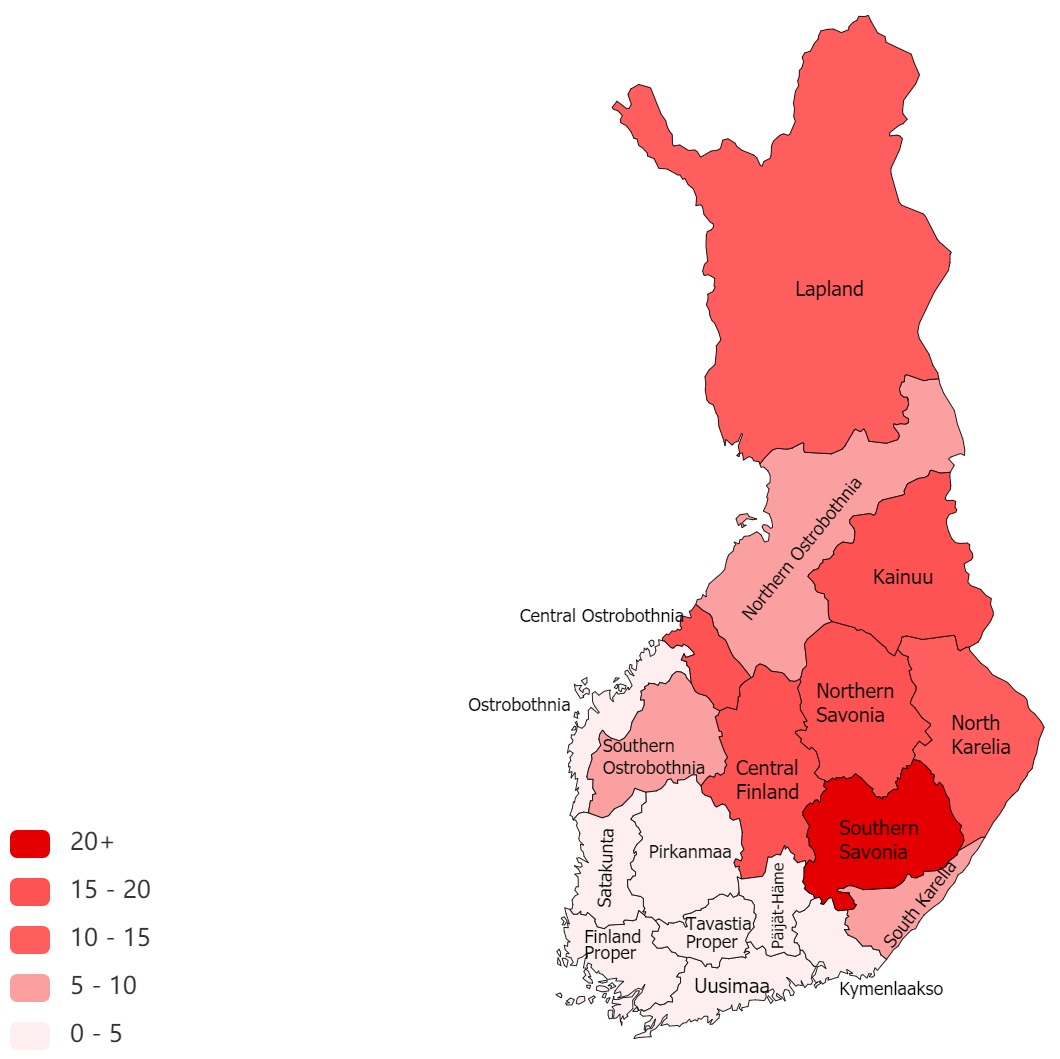

Supplement: Supplementary file 1 [file inf-41-e113-s001.docx]
