# Supplementary material for: Changes in the Epidemiology of Zoonotic Infections in Children: A Nationwide Register Study in Finland
Source: Pediatr Infect Dis J. 2021 Dec 28;41(4):e113–9. doi: 10.1097/INF.0000000000003440 (PMC8920006; doi:10.1097/INF.0000000000003440)
Supplement: Supplementary file 2 [file inf-41-e113-s002.docx]

**
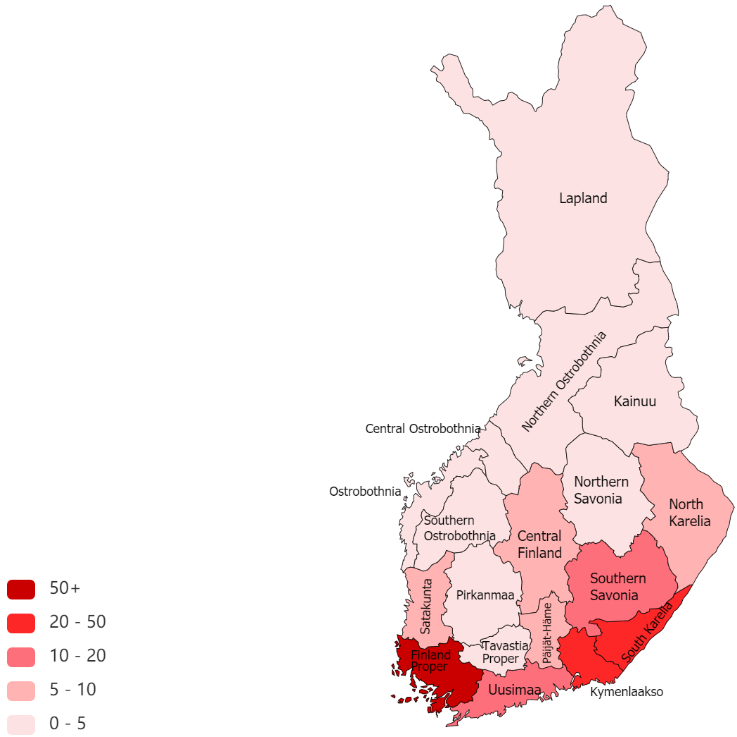

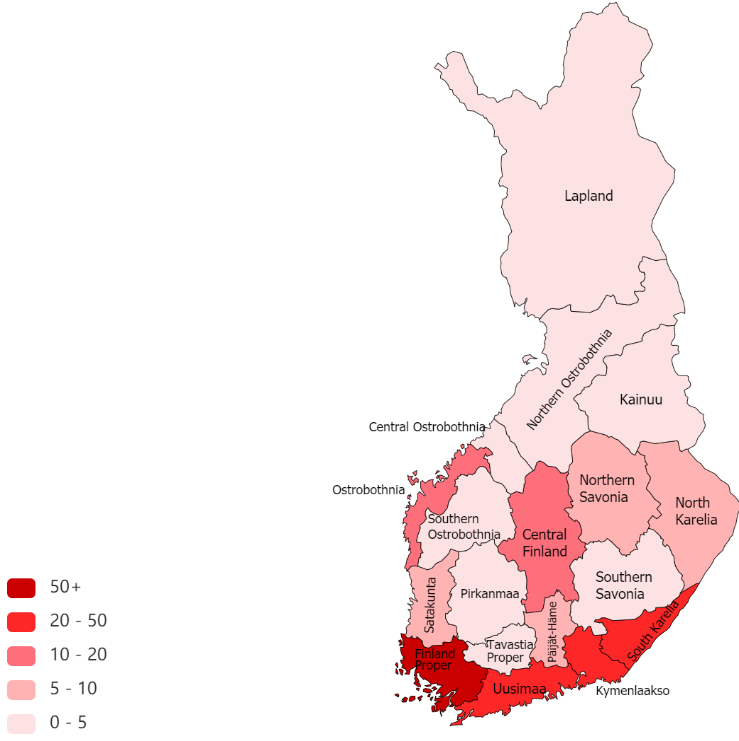

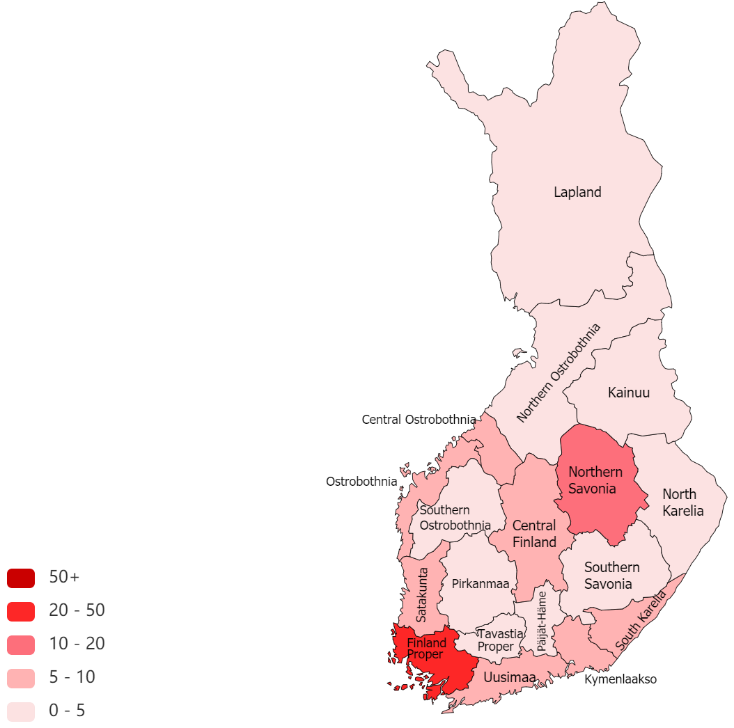
Supplemental Digital Content 2.** Geographic incidences of *Borrelia burgdorferi* infections A) from 1996 to 2003, B) from 2004 to 2011, and C) from 2012 to 2019 per 100,000 person years.

A) B) C)
