# Supplementary material for: Changes in the Epidemiology of Zoonotic Infections in Children: A Nationwide Register Study in Finland
Source: Pediatr Infect Dis J. 2021 Dec 28;41(4):e113–9. doi: 10.1097/INF.0000000000003440 (PMC8920006; doi:10.1097/INF.0000000000003440)
Supplement: Supplementary file 3 [file inf-41-e113-s003.docx]

**Supplemental Digital Content 3.** Yearly mean temperature deviation from 1996 to 2019 in Finland. The deviation is calculated from the average temperature of climate years 1980–2010 in three different geographic regions of Finland.
